# The Evolution of Integrated Chronic Disease Prevention in Alberta, Canada

**Published:** 2006-06-15

**Authors:** Kim Raine, Sharlene Wolbeck Minke, Ernest Khalema, Cynthia Smith, Ronald C Plotnikoff

**Affiliations:** Centre for Health Promotion Studies. Dr Raine is also affiliated with the Alberta Heart Health Project, University of Alberta, Edmonton, Canada; Alberta Heart Health Project, University of Alberta, Edmonton, Canada; Alberta Heart Health Project, University of Alberta, Edmonton, Canada; Alberta Heart Health Project, University of Alberta, Edmonton, Canada, and Centre for Health Promotion Studies, University of Alberta, Edmonton, Canada; Alberta Heart Health Project, University of Alberta, Edmonton, Canada, and Centre for Health Promotion Studies, University of Alberta, Edmonton, Canada

## Abstract

**Background:**

Recognition of the common risk factors for leading chronic diseases in Canada has contributed to the development of integrated chronic disease prevention and health promotion approaches. The Alberta Heart Health Project studied the capacity of health organizations in Alberta, Canada, to engage in heart health promotion. This article describes how the Alberta Heart Health Project acted on emerging research findings describing the preliminary stages of integrated chronic disease prevention in Alberta to provide leadership to encourage provincial chronic disease prevention efforts.

**Context:**

Political support for integrated chronic disease prevention was evident at the provincial and federal levels in Canada. As a result of organizational restructuring, loss of key health promotion champions, and decreased funding allocations, Alberta's regional health authorities sought increased efficiency in their chronic disease prevention efforts.

**Methods:**

Descriptive data were derived from a brief questionnaire on regional health authorities' chronic disease prevention priorities and activities, an inventory of regional health authority health promotion programs and services, content analysis of key regional health authority documents, and focus groups with regional health authority staff, management, and policymakers.

**Consequences:**

In 2002, the Alberta Heart Health Project data revealed that many regional health authorities were beginning to engage in integrated chronic disease prevention. However, little collaboration occurred across the health organizations; provincial leadership to facilitate collaboration and networking for integrated chronic disease prevention was needed..

**Interpretation:**

Results supported the growing momentum for provincial leadership to enhance collaboration for integrated chronic disease prevention, which contributed to the development of the Alberta Healthy Living Network. The government's assistance is also needed to support the intersectoral collaborations essential for integrated chronic disease prevention.

## Background

Recognition of the common risk factors for the leading chronic diseases in Canada has contributed to the development of integrated chronic disease prevention (CDP) approaches within the health promotion paradigm. The Alberta Heart Health Project (AHHP), which is part of the Canadian Heart Health Initiative (CHHI), studied capacity of health care organizations (regional health authorities [RHAs]) in Alberta, Canada, to engage in heart health promotion. This case study describes how the AHHP acted on emerging research findings describing the preliminary stages of integrated CDP in Alberta to provide leadership in moving provincial CDP efforts forward.

In Canada, chronic diseases such as cardiovascular disease, cancer, diabetes, and chronic obstructive pulmonary disease (COPD) are the leading causes of morbidity and mortality (1-4). In addition to their effects on individuals and their families, the treatment of chronic diseases requires significant health care resources. An Alberta analysis of mortality and morbidity data from 2000 revealed that an individual with a chronic disease consumes 10 times more health care resources than does an individual with no evidence of chronic disease. The report also determined that in the same year, the cost of providing treatment (i.e., physician and hospital services) to individuals with more than one chronic disease "result[ed] in a high overall economic burden ($137 million)" to the province (3).

Traditionally, chronic disease prevention efforts have been largely focused on specific diseases, such as heart disease or diabetes. However, there is growing recognition that the risk factors are similar for cardiovascular disease (CVD), type 2 diabetes, COPD, and some cancers. The shared behavioral risk factors are tobacco use, unhealthy diet, and sedentary lifestyle (5). Canadian statistical data reveal the levels of health risk posed by each of these factors. In 2003, 21% of women and 17% of men (aged 18 years and older) reported that they were smokers (6). Obesity increased among Canadian adults (aged 18 and older) by 66% (from 14% to 23%) between 1978-1979 and 2004 (7). Although an increase in moderate physical activity was evident among Canadians with acceptable body weight (i.e., 38% in 1994–1995 compared with 43% in 2000–2001), little change occurred in the physical activity of obese Canadian adults; 33% engaged in moderate physical activity in 1994 to 1995 compared with 34% in 2000 to 2001 (8). It is expected that if the current level of risk factors remains unchanged, the aging Canadian population will experience even greater health and social costs from chronic disease (9).

### Integrated chronic disease prevention

Recognition of the preventable risk factors shared by leading chronic diseases in Canada is shifting the field of health promotion toward integrated CDP. The multiplicity and complexity of an integrated CDP approach is articulated in the following definition formulated by Shiell (10) in his synthesis research:

[Integrated chronic disease prevention is an approach] . . . that targets *more than one risk factor or disease outcome, more than one level of influence, more than one disciplinary perspective, more than one type of research method, or more than one societal sector,* and which targets populations — rather than individuals — as a unit.

Key concepts in the integrated CDP model include an ecological perspective, intersectoral action, multilevel intervention, and collaborative processes. The influence of the ecological perspective is evident in that integrated CDP frameworks consider the interdependence between individuals and the broader socioenvironmental context (11,12). Accordingly, the scope of analysis extends beyond individual and biomedical factors to the population level. It is at the population level that the links between individual lifestyle behaviors and the social determinants of health, such as education, employment and working conditions, culture, income, and social supports, are explicated (11,13).

Although the health sector is called on to provide leadership for integrated CDP, effective action requires intersectoral involvement (14). Sectors that are not traditionally involved in health initiatives, such as transportation, housing, and education, have critical roles to play in integrated CDP. Furthermore, participation must include public and private sector organizations (10,15). It is believed that pooling expertise, influence, and perspectives creates a synergy that achieves results greater than those possible by a single organization or sector (15-18).

Collaboration, partnerships, and networking are intrinsic to integrated CDP (9,10,14). Collaborative processes can strengthen existing networks and partnerships and also may encourage the development of new links among organizations from various sectors and levels (i.e., local, regional, provincial, and national) (9,10,14). As such, collaborative processes enhance the coordination of CDP efforts, lessen duplication, and "facilitate the development and implementation of policy" (10). It follows that developing and sustaining collaborative processes and networks are priority strategies for an integrated CDP approach (9).

### AHHP

The AHHP began with a provincial heart health survey that revealed high rates of cardiovascular disease risk factors among Albertans (19). During the demonstration phase (1993–1998), the AHHP sought to understand the effectiveness of community-based approaches to heart health promotion. The organizational capacity of RHAs to engage in heart health promotion was explored in the dissemination phase (1999–2005). This research project was grounded in the capacity-building model informed by Rogers' diffusion of innovation theory (20). *Capacity building* was defined as the capability of an organization to promote health as formed by the dynamic interaction of organizational leadership, will to act, and infrastructure (21,22). Early in this investigation of heart health promotion capacity, we recognized that RHAs were increasingly shifting to an integrated CDP approach in their health promotion and CDP activities.

## Context

Support for integrated chronic disease prevention in Canada is apparent on multiple fronts. Recent federal and provincial government reports on health care, such as *A Framework for Reform: Premier's Advisory Council on Health* (23) and *The Health of Canadians* — *The Federal Role* (24), provide a political base of support. Although these reports call for increased attention to the prevention of chronic disease, the importance of a national integrated CDP strategy is specifically identified in* The Health of Canadians* — *The Federal Role*. The rise of integrated CDP is also amply discussed in Canadian and international conference proceedings (e.g., Canadian Heart Health Network Workshop, 2002, Moving from Heart Health to Chronic Disease Prevention) as well as technical reports (e.g., Chronic Disease Prevention Alliance of Canada [available from: www.chronicdiseaseprevention.ca/], Ontario Health Promotion E-Mail Bulletin [available from: www.ohpe.ca/ebulletin/]).

In 1993 and 1994, reform of the Alberta health care system led to the centralization of acute care, long-term care, and community health services within 17 RHAs. Provincial restructuring in 2003 altered the original geographic boundaries and reduced the number of RHAs to nine. This reorganization contributed to a loss of health promotion champions in organizational cultures that were dominated by acute care services and biomedical values (25). In an environment of constant change and reduced funding allocations, the RHAs searched for increased efficiencies in their health promotion and CDP approaches.

## Methods

The AHHP studied the capacity of RHAs to engage in health promotion and CDP through a mixed-method approach within a time-series research design. Between 2000 and 2004, the RHAs' organizational capacity for health promotion was assessed annually through a validated survey. Beginning in 2001, assessments (e.g., focus groups, interviews) were conducted annually in three RHAs of varying capacity levels. In 2002 and 2003, the AHHP team also conducted qualitative analyses of organizational documents and a survey of RHA health promotion and CDP programs. This article reports on a cross-section of these available data at a time when a move toward chronic disease prevention was beginning within the RHAs.

### Organizational capacity survey

The Health Promotion Organizational Capacity Survey was developed to assess the key dimensions of the RHAs' organizational capacity (i.e., infrastructure, will, and leadership) at individual and organizational levels (26-29). To develop the survey, the AHHP team initially met with CHHI researchers from across Canada to learn about the capacity assessment tools used in other projects (i.e., Saskatchewan, British Columbia, Ontario, and Manitoba). The initial draft of the survey incorporated the CHHI group experiences and key information from current research literature. The content validity of the instrument was established and verified by an expert opinion focus group. Following revisions to incorporate the focus group results, the survey was pilot tested with a representative sample of respondents from local, provincial, and national organizations that were not involved in the study. The purpose of the pilot test was to verify the clarity of concepts and questions in the survey. This draft was also reviewed by 37 CHHI researchers to confirm content validity. The final version of the survey incorporated the feedback from the pilot test and CHHI researchers. For more information on survey psychometrics, see various AHHP publications (26-29).

All 17 RHAs were invited to participate in the study, and none refused. Site coordinators in each RHA recruited study participants from three levels of the organization (service providers, management, and board members) and administered the survey. The AHHP provided the site coordinators with survey packages for each participant. The packages consisted of the survey, a return envelope, project information sheets, and consent forms.

In 2000, 144 participants in 17 regions completed the survey. In accordance with the time series design, RHAs were resurveyed in 2001 (n = 122 with 17 RHAs), 2002 (n = 90 with 16 RHAs) and 2004 (n = 72 with 9 RHAs). The survey was not administered in 2003 because of the provincial restructuring of RHAs.

Results from the 2000 and 2001 surveys identified a change in the RHAs' health promotion activities in that many did not exclusively focus on heart health promotion (data not shown). To explore this change more fully, additional questions focusing on integrated CDP were added to the annual 2002 survey (CDP survey insert, Table 1). In accordance with the preliminary nature of integrated CDP reflected in the previous survey results, the operational definition used on the 2002 survey insert focused on the integration of multiple diseases (e.g., cancer, CVD, diabetes) and multiple modifiable risk factors (inadequate nutrition, physical inactivity, and tobacco use) that apply to CVD as well as cancer, diabetes, and other chronic disease risk factors. Finally, the following open-ended question provided the opportunity for respondents to provide more detail and context: "Please comment on the extent to which CDP is integrated into your organization, and on how much of the CDP is related to heart health promotion." In 2002, 82 of the 90 respondents chose to complete the CDP insert, but not all of the respondents replied to each question (Table 1). The CDP survey inserts are most relevant to this study because they represent the first time integrated CDP was specifically investigated in Alberta. The cross-sectional data gathered in the CDP survey insert were analyzed using descriptive statistics (i.e., frequencies) to characterize respondents' perceptions of the status of CDP activities in the RHAs.

### Document review

A review of the RHAs' key documents provided information about the extent to which the organizations were actively involved in health promotion, the level of corporate support for health promotion, and the evolution of CDP. The research team systematically reviewed the content of RHA business plans, annual reports, and other strategic health promotion reports. Thematic content analysis of reports was used to identify and characterize public representations of health promotion and CDP priorities, health promotion activities, programs, services, policies, and resources in the areas of chronic disease prevention and heart health promotion.

### Inventory of RHA programs

Because not all health promotion activities are evident within readily accessible RHA documents, a systematic inventory of heart health promotion initiatives and programs within and outside the RHAs was undertaken in 2002 and 2004. This information provided context for the organizational capacity survey results and indicated the level of collaboration and integration across Alberta. Key informants in each of the 17 RHAs were consulted about program activities in the areas of tobacco, nutrition, physical activity, and other social determinants of health. Respondents also provided the research team with supporting program information that clarified specific questions and programs. These additional documents were reviewed for content. For the purpose of this case study, exemplary results such as the tobacco reduction initiatives from the 2002 inventory are included (Table 2) because they provide temporal context and describe the state of RHA health promotion and CDP initiatives at the beginning of the shift to integrated CDP.

### Intensive assessments

Based on the results of the baseline organizational capacity survey from 2000, each of the 17 RHAs was categorized as having low, medium, or high capacity for health promotion according to tertile scores for overall capacity. The research team then selected one RHA from each capacity category (total of three) for intensive assessment during the remainder of the study. The RHAs were selected according to geographical variability (representing northern, central, and southern Alberta), no previous participation in the AHHP, and willingness to participate in the assessments.

### Interviews and focus groups

The core of the intensive assessments was interviews and focus groups with key informants, who most often were also survey respondents. In 2001, 24 semistructured interviews were conducted with the three RHAs. Focus groups conducted in 2002 and 2003 in each of the three RHAs were attended by between 5 and 12 participants. In each focus group, participants included board members, senior and middle managers, and front-line staff. Questions prompted participants to reflect on what facilitated organizational health promotion capacity in their RHA, what was needed for capacity building, and which incentives were helpful for capacity building. The focus groups were recorded and transcribed verbatim.

Content and thematic analyses procedures (30) were used for the qualitative aspect of the survey (open-ended questions), document review, and intensive assessments.

Data interpretation involved integrating qualitative and quantitative findings. For example, quotes from focus groups were used to provide context for the descriptive statistical data derived from the CDP survey insert; the inventory of RHA health promotion programs was used to corroborate qualitative data from interviews.

## Consequences

### Integrated CDP within RHAs

The organizational capacity survey data indicated that by 2002, most RHAs were already using a CDP approach. More than half (59%) of the respondents indicated that their organization had a CDP champion (Table 1). Most respondents (91%) also reported that their organization's CDP approach involved heart health. In addition, it was clear that CDP and health promotion were becoming increasingly integrated throughout the health organizations (56%). The integration of CDP and health promotion across multiple disciplinary perspectives was aptly described by focus group participants:

So we're trying to enmesh prevention promotion among our acute care population as much as we can. (High-capacity region focus group, 2003)

I don't think health promotion is any department's job or any one person's job; I think it's a philosophy and that it should be involved in everybody's health promotion. I mean, public health is doing health promotion every day of the week and every interaction that they have, as is cardiac rehab and as is diabetes. (Low-capacity region focus group, 2003)

### The gap: lack of collaboration

Although individual RHAs were increasingly engaged in integrated CDP, program survey results revealed a lack of collaboration across the RHAs' tobacco reduction initiatives (Table 2). The titles that described the initiatives, which were often developed in similar time frames, implied that each RHA was developing unique initiatives to address common population health risks. For example, in 2000, 9 of the 17 RHAs described similar community-based tobacco cessation initiatives that were specific to their region. Focus group participants also indicated an awareness of the multiple initiatives among the RHAs and articulated a need for increased collaboration:

[T]hrough our Action for Health portfolio [provincial funding program], there's all kinds of interesting things we do, and all of it's written up in reports. Yet at no time ever does anyone come back and say, "I read about what you're doing; I read your report. Tell me more. Tell me — so-and-so is doing something similar; is there some way we can bring the best of those together?" It's like whatever you do locally seems to just get stuck there. So I don't know; fill me in, but is there a provincial structure that is geared towards finding the best of the best, and actually maximizing those early interventions to make them something bigger? (Medium-capacity region focus group, 2003)

We have to work just a little harder to really communicate and to make sure it's really that global thinking again, thinking past boundaries of your own department, your own site, your own building, your own community. (Low-capacity region focus group, 2003)

 I think if you're going to do a program like Heart Health, I think it needs to be — everybody in the province should probably be doing similar strategies, or even a number of provincial strategies. (High-capacity region focus group, 2003)

. . . I wish they would work more together and have more provincial resources. . . . (Medium-capacity region focus group, 2003)

### The need: leadership for collaboration

Program survey results also suggested that collaboration occurred among the RHAs with provincially led campaigns such as the Clean Air Campaign. Focus group participants called for provincial leadership to facilitate collaboration on integrated CDP and health promotion strategies. It was noted that the provincial department of health had previously played this role:

. . . I think preregionalization, so say pre-1995, the health units had a mandate to do health promotion. . . . There might be injury prevention, there might be a heart health project, but the difference was that the province provided more leadership. The province often developed the programs and the materials and everything else, and we were able to pick up on that. . . . (Medium-capacity region focus group, 2003)

A sense of timeliness was conveyed by focus group participants. They believed that political and organizational will existed for the increased collaboration and networking intrinsic to an integrated CDP approach in Alberta:

 I think that the hill that we're climbing in terms of change, that the steepness of the slope is really decreasing because of the . . . network with other areas across the province and across the country, and recognize that we're not climbing this hill alone, we're all climbing this hill, and it really makes every step easier. I think that this is a time right now where people are forging new connections. . . . But when we understand that everybody is trying to change this way, and it's long-term and worth the investment, it just makes it easier to go down that route. (Medium-capacity region focus group, 2003)

So Alberta Health has been definitely supportive in saying, "We're going to help you create a network across the province so that you can benefit from that and be moving together in the same direction." (Medium-capacity region focus group, 2003)

## Interpretation

### AHHP leadership

In 2002, the AHHP team acted on these preliminary research results, which described the need for provincial leadership to enhance collaboration for integrated CDP in the province. The AHHP was an instrumental partner in organizing the forum Preventing Chronic Disease: Working Together in an Integrated Approach. Its purpose was to initiate a critical discussion about "the need for provincial leadership and commitment to health promotion and chronic disease prevention" (15). Stakeholders from the 17 RHAs, governmental departments of health and wellness (provincial and federal), nongovernmental organizations, and organizations beyond the traditional health sector were invited and participated in the forum. The momentum generated at the forum contributed to the formation of the Alberta Healthy Living Network (AHLN) as the next step in the evolution of integrated CDP in Alberta.

### AHLN

The AHLN reflects the intrinsic complexity of integrated CDP approaches. The mission is "to provide leadership for integrated, collaborative action to promote health and prevent chronic disease in Alberta" (15). The network is philosophically grounded in health promotion principles, and its strategies occur at the population level. AHLN members are deliberately recruited from organizations at the local, regional, and provincial levels across Alberta. Furthermore, the AHLN actively links existing provincial (e.g., Alberta Diabetes Strategy), national (e.g., Chronic Disease Prevention Alliance of Canada), and international (e.g., Countrywide Integrated Noncommunicable Disease Intervention [CINDI] — World Health Organization) healthy living and CDP strategies. Network members are from the traditional health sector as well as nontraditional sectors instrumental to health promotion and CDP, such as education, transportation, active living, and recreation. Since its inception in November 2003, the AHLN's membership has grown to 141 member organizations and coalitions. The AHLN facilitates the collaboration and networking that are essential to integrating CDP in Alberta.

The AHLN's Alberta Healthy Living Framework (15) facilitates the operationalization of integrated CDP in Alberta by identifying seven priority strategies for action on chronic disease in Alberta (Table 3). Each priority strategy is evidence based and has specified outcomes and actions. At this time, the areas of focus target the common chronic disease risk factors of healthy eating, active living, tobacco reduction, mental health, and injury prevention (15). The AHLN has also provided leadership for integrated CDP by taking action on several of the priority strategies (Table 3). These activities are expected to contribute to the science and practice of integrated CDP in Alberta.

It is clear that chronic diseases have a substantial impact on Canadian society. The need to increase the efficiency and efficacy of traditional, single-disease–focused prevention efforts has resulted in the growth of integrated CDP approaches. Alberta RHAs were in the preliminary stages of integrated CDP in 2002 but needed provincial leadership to enhance multilevel and intersectoral collaboration and networking. As the priority strategies described in the Alberta Healthy Living Framework are realized, the AHLN will have a leadership role.

Although enhanced collaboration and networking among the RHAs are excellent midpoint objectives, structural factors (e.g., political, financial) must be in place for interventions to be implemented and thoroughly evaluated (10). It follows that government support of integrated approaches to addressing chronic disease is necessary. Policies and program funding that target the full range of determinants of health must be collaboratively developed by government departments such as health, education, and human resources and employment. In addition, the voluntary, professional, academic, and private sectors contribute to action on the root causes of health. Because the most important level of integration is the level of conceptualizing and planning (31), effective leadership is important. The public health system, the embodiment of our publicly funded organized efforts to prevent disease and promote health, must take responsibility for facilitating the intersectoral collaborations fundamental to integrated chronic disease prevention (31).

## Figures and Tables

**Table 1:** Respondents' Perceptions of Chronic Disease Prevention in Regional Health Authorities, Alberta, Canada, 2002 (N = 90^a^)

**Survey Question and Response**	**Frequency, No. (%)**
Is there a chronic disease prevention champion in your organization? (n = 63)	
Yes	37 (58.7)
No	14 (22.2)
Don't know	12 (19.0)
Is heart health promotion part of the chronic disease prevention focus in your region? (n = 63)	
Yes	57 (90.5)
No	6 (9.5)
Is chronic disease prevention (promotion) integrated throughout the organization (for example, is it part of the organization's business plan, part of job descriptions across the organization)? (n = 55)	
Yes	31 (56.4)
No	24 (43.6)

a Not all of the 90 respondents to the 2002 Health Promotion Organizational Healthy Capacity Survey chose to complete these additional questions about chronic disease prevention.

**Table 2:** Tobacco Reduction Activities in Regional Health Authorities (RHAs), Alberta, Canada, 2000 and 2002

**Target Strategies**	**Initiative**	**Number of RHAs Participating**	

		**2000**	**2002**
General provincial strategies	Clean Air Campaign — Smoke-Free Homes	6	—
	Clear the Air Campaign; Weedless Wednesday; World No Tobacco Day	1	—
	Partnership With Alberta Alcohol and Drug Abuse Commission	—	1
	Partnership With Alberta Tobacco Reduction Alliance	7	1
	Partnership With Action for Health	1	—
	Working with provincial and community partners for tobacco reduction initiatives	1	—
Maternal–child strategies	Assist pregnant women to stop smoking	—	1
	Breathing for Two	1	—
	Clinical training for tobacco reduction among pregnant women	1	—
	Food for Two (prenatal awareness of tobacco risks)	1	—
	Healthy Moms/Healthy Babies	1	—
	Kick Butt for Two	—	1
	Maternal–child quality management team	1	—
	Prenatal nutrition program (emphasis on tobacco reduction)	—	1
Youth initiatives	BLAST (youth creating plans to address tobacco issues)	2	—
	Expand initiatives with youth emphasis	—	1
	Flight Path Youth Tobacco Reduction conference	1	—
	Peace Adolescent Health	—	1
	Reduce youth tobacco use	—	1
	Teen Health Survey	1	—
	Youth tobacco cessation programs	2	—
School-based initiatives	Collaborate with school division	1	—
	CANOE (Ceasing Addiction to Nicotine with Others' Encouragement), high school	1	—
	Interactive kit for teachers	—	1
	School education programs, grades 3–7	—	1
	School grants for tobacco cessation activities	1	—
	Students funded to attend peer-support and leadership conference	—	1
	Tobacco Addiction Awareness Prevention program	1	—
	Tobacco reduction initiatives in schools	—	1
	Youth tobacco survey, junior and senior high schools	1	—
Community-based initiatives	Activities of Chinook Tobacco Resource Network	1	—
	Adult tobacco cessation programs	2	—
	Catching Our Breath	—	1
	Freedom From Smoking	—	1
	Harm reduction approach	—	1
	Increase the number of smoke-free environments (e.g., in home and community settings)	1	2
	Kick Butt Campaign	1	—
	Kic the Nic	1	—
	OFF Your Butts	1	1
	Smoking cessation information on Web site	—	1
	Smoke-Free Bow Valley	—	1
	Smoke-Free Registry for Business in David Thompson Health Region (online)	1	—
	Smoke Talk	1	—
	Start Stopping (smoking cessation program)	1	—
	Teaming Up for Tobacco-Free Kids	—	1
	Tobacco Addiction Awareness Prevention program	1	—
	*Tobacco-Less Times* (newsletter)	1	—
	Tobacco reduction activities (unspecified)	2	—
	Tobacco reduction coalitions	2	—
	Tobacco reduction environmental scan and operational plan completed	1	—
	Toxic Tunnel	1	1
	Tobacco Reduction Committee (partnered with community groups)	1	—
	*Your Guide to a Smoke-Free Future*	—	1
Organizational initiatives	Dental Health Services integrated tobacco reduction	1	—
	Smoke-free environment in and on regional health authority property	3	—
	Workplace wellness program (smoking cessation)	—	1
Policy initiatives	Advocacy activities (unspecified)	1	—
	Smoking control policy for regional health authority facilities	1	2
	Smoke-free plebiscite	—	1
	Policy advocacy for decreased smoking rates (i.e., city bylaw review)	1	1

**Table 3:** Alberta Healthy Living Network's Seven Priority Strategies and Related Actions

**Priority Strategy**	**Purpose (15)**	**Action**
Partnership development and community links	Strengthen partnerships and enable coordinated mobilization of resources in the community	Baseline interorganizational relationships among AHLN members documented through network mapping research (2004) Public Health Agency of Canada (Population Health Fund) funding provided to three communities to facilitate network development Commissioned report *Alberta Healthy Living Network: Network Development Strategy* (2005)
Awareness and education	Facilitate coordinated information and education for healthy living in Alberta	Cosponsored forum in May 2004: Collaborative Action for Healthy Living: It Takes Us All; 131 participants
Surveillance	Advocate for and support development of surveillance systems for chronic diseases and risk factors	Working group determined action
Best practices	Establish a system that facilitates sharing of evidence-based practice for policies and programs that address the population-based risk factors and underlying determinants of health for health promotion and chronic disease prevention	Commissioned report *Chronic Diseases in Alberta: Cost of Treatment and Investment in Prevention* (2005)
Research and evaluation	Collaborate on health promotion and chronic disease prevention research and evaluation initiatives	Developed evaluation framework to assess impact of AHLN Commissioned process evaluation of AHLN structures and processes (2005)
Health disparities	Increase opportunities for healthy living among underserved groups in Alberta	Working group determined action
Healthy public policies	Advocate for and create healthy public policies	Working group determined action
